# A Review of Wastewater-Based Epidemiology for the SARS-CoV-2 Virus in Rural, Remote, and Resource-Constrained Settings Internationally: Insights for Implementation, Research, and Policy for First Nations in Canada

**DOI:** 10.3390/ijerph21111429

**Published:** 2024-10-28

**Authors:** Jessica Annan, Rita Henderson, Mandi Gray, Rhonda Gail Clark, Chris Sarin, Kerry Black

**Affiliations:** 1 Department of Family Medicine, Cumming School of Medicine, University of Calgary, 3330 Hospital Drive NW, Calgary, AB T2N 4N1, Canada; jcannan@ucalgary.ca (J.A.); rihender@ucalgary.ca (R.H.); 2 Department of Sociology, Trent University, Oshawa, NG11 8NS, Canada; mandigray@trentu.ca; 3 Department of Biological Sciences, Geomicrobiology Group, University of Calgary, Calgary, AB T2N 1N5, Canada; rgclark@ucalgary.ca; 4 Indigenous Services Canada, First Nations and Inuit Health Branch, Alberta Region, Canada Place, Suite 730 9700, Jasper Avenue, Edmonton, AB T5J 4C3, Canada; chris.sarin@sac-isc.gc.ca; 5 Department of Civil Engineering, Schulich School of Engineering, University of Calgary, 2500 University Drive NW, Calgary, AB T2N 1N4, Canada

**Keywords:** wastewater surveillance, COVID-19, First Nations, public health, equity, implementation readiness, vulnerable communities

## Abstract

Wastewater-based epidemiology (WBE) is regarded as a support tool for detecting and assessing the prevalence of infectious diseases at a population level. For rural, remote, and resource-constrained communities with little access to other public health monitoring tools, WBE can be a low-cost approach to filling gaps in population health knowledge to inform public health risk assessment and decision-making. This rapid review explores and discusses unique considerations of WBE in key settings, with a focus on the detection of the SARS-CoV-2 virus, which has rapidly expanded WBE infrastructure globally. To frame our understanding of possibilities for WBE with First Nations in Alberta, we address the following questions: What are the unique considerations and challenges for WBE under similar contexts in rural, remote, or resource-constrained settings? What are the resources and expertise required to support WBE? This review identifies several unique considerations for WBE in rural, remote, and resource-constrained communities, including costs, accessibility, operator capacity, wastewater infrastructure, and data mobilization—highlighting the need for equity in WBE. In summary, most resource-constrained communities require additional support from external research and/or governmental bodies to undertake WBE.

## 1. Introduction

The COVID-19 pandemic has highlighted the importance of wastewater-based epidemiology (WBE) in tracking and understanding infectious diseases worldwide. Before the COVID-19 pandemic, WBE had been used to monitor the population prevalence of enteric viruses, such as hepatitis A, norovirus, rotavirus, and polio [1]. Since 2020, numerous university-based teams across Canada have set up and successfully run various programs to monitor for the genetic markers of SARS-CoV-2 in wastewater, providing important additional information to overall COVID-19 testing. In addition, Statistics Canada and the Public Health Agency of Canada (PHAC) have collaborated on the Canadian Wastewater Survey (CWS) to conduct WBE in various wastewater treatment plants (WWTPs) in five municipalities across Canada (Halifax, Montreal, Toronto, Edmonton, and Vancouver) for the detection of the SARS-CoV-2 virus and variants of concern (VOC). Further, PHAC is working in collaboration with other federal departments, provincial, territorial, and municipal governments, as well as universities throughout Canada toward creating a pan-Canadian WBE network for monitoring the SARS-CoV-2 virus [2]. As of January 2023, this network of federal, provincial, territorial, and academic WBE teams provides coverage for approximately 64% of the total population of Canada [3]. Since 2020, this network has also collaborated as the Canadian COVID-19 Wastewater Coalition, launched by the Canadian Water Network to exchange knowledge on WBE to validate pro-ject results, establish best practices, and ensure results are beneficial to public health actors and communities [4].

Despite the value of WBE for public health decision-makers, it has not been implemented on the same scale across First Nations in Canada. This is notable in the western province of Alberta, where health services and monitoring in many First Nations are resource-constrained, such that they lack the capacity for mass clinical testing and face logistical challenges arising from the geographical dispersion of community members in rural and remote areas. To fill gaps in knowledge on community-level health, WBE has been advocated as a relatively low-cost approach to monitor population health and inform public health policy [5,6,7,8,9,10,11,12,13,14,15,16,17,18,19,20,21,22,23,24,25,26,27,28].

This rapid review focuses on the unique challenges and opportunities for WBE implementation in settings with similar geographic, infrastructural, and human resources constraints. We focus on the detection of SARS-CoV-2 in particular as a virus that has rapidly expanded possibilities in the area of WBE. The purpose of this review is to gather insights about how communities can implement WBE and track population-level data on the spread and prevalence of pathogens or other health indicators to make public health decisions. This review is part of a larger Pan-Alberta WBE research project that also seeks to critically examine whether and how researchers, healthcare organizations, and leaders can support First Nations in building capacity for wastewater-based epidemiology in general.

There are 138 reserves in Alberta, each affiliated with one of the province’s 46 First Nations, all of which possess unique geographic, cultural, or other contextual factors that may facilitate or change environmental surveillance. Many of these First Nations communities are remote and rural and contend with limitations of constrained resources. After a preliminary search, it was determined that there is a dearth of literature specific to WBE on First Nations specifically and rural/remote contexts generally. To facilitate further insights into the factors that shape WBE on First Nations in Alberta, we expanded our scope to explore challenges, opportunities, barriers, and facilitators to WBE in several international settings with comparable characteristics. These settings included remote and/or rural communities and resource-constrained and/or low-income communities.

To frame our understanding of the potential of WBE in First Nations in Alberta, this review sought to address the following questions: What are the unique considerations and challenges for WBE in such settings? What capacity-building measures are necessary to implement WBE in these communities?

## 2. Methods

A rapid review of peer-reviewed literature was conducted following the guidelines set by Canada’s National Collaborating Centre for Methods and Tools [29]. Before beginning the search, the Cochrane Database of Systematic Reviews was checked for pre-existing reviews on the topic of WBE on First Nations or Native American reservations, and then for use of the method for detecting COVID-19 in rural or remote areas; no such reviews were found. Considering this, criteria for literature inclusion and exclusion were established to capture special considerations of WBE in remote and/or rural communities and resource-constrained and/or low-income contexts. This criterion was conceptualized as First Nations communities in Alberta are often geographically rural and/or remote, which presents unique considerations for sampling protocols and laboratory access. Additionally, due to the historical and continued impacts of colonization, socio-economic marginalization is a factor in conducting WBE in First Nations communities. However, a critical point to address is that these extrapolations are not to conflate these geographic and economic factors with the socio-political and cultural nuances that shape research with First Nations in Canada. Rather, this review aims to gather insights that might inform work with First Nations in Alberta, considering the dearth of literature on WBE in this area.

### 2.1. Rapid Review

The initial search terms were chosen in consultation with the multi-disciplinary research team of the larger Pan-Alberta WBE project that inspired this review. Additional terms were included by identifying keywords associated with the initial articles collected during the testing of the search strategy. Between early November and late December 2022, Medline, EMBASE, Social Sciences Citation Index, SCOPUS, JSTOR, and the first 100 results of Google Scholar were searched using the following search terms in assorted combinations (Table 1). The search was updated in September 2024.

Because of the novel nature of the topic, no period was specified for the inclusion of literature. As our interest is drawn to WBE for the detection of the SARS-CoV-2 virus, it is not surprising that the majority of studies included were published since 2020.

### 2.2. Inclusion and Exclusion Criteria

Literature was included if it satisfied the following criteria: (i) primary and secondary empirical studies (including qualitative, quantitative, or mixed-method studies; reviews or summaries of empirical studies; policy briefs and analyses; and implementation studies); (ii) focusing on WBE and the detection of infectious diseases and other health indicators; (iii) set in, or referring to remote and/or rural communities or resource-constrained and/or low-income communities; and (iv) full text available in English.

Literature was excluded if it met the following criteria: (i) containing only abstracts without full text available; (ii) including conference abstracts; (iii) not available in English; and (iv) focusing on large urban or peri-urban areas without documenting resource constraints.

### 2.3. Data Extraction and Analysis

One reviewer searched databases for studies which were uploaded to Covidence. Two reviewers performed title and abstract screening and full-text review. One reviewer undertook data extraction and synthesis. The extraction inventory included setting, resources needed, perceived benefits of WBE, methodology, reported implications and mobilization of WBE data for communities, and logistical considerations.

The reference lists for all eligible studies were also screened for relevant studies that were not found via database and Google searches. This hand search resulted in the addition of four articles.

## 3. Results

We initially identified 31 studies that discussed WBE in remote and/or rural communities, or resource-constrained and/or low-income communities. The updated search retrieved 15 additional studies.

**Figure 1:**
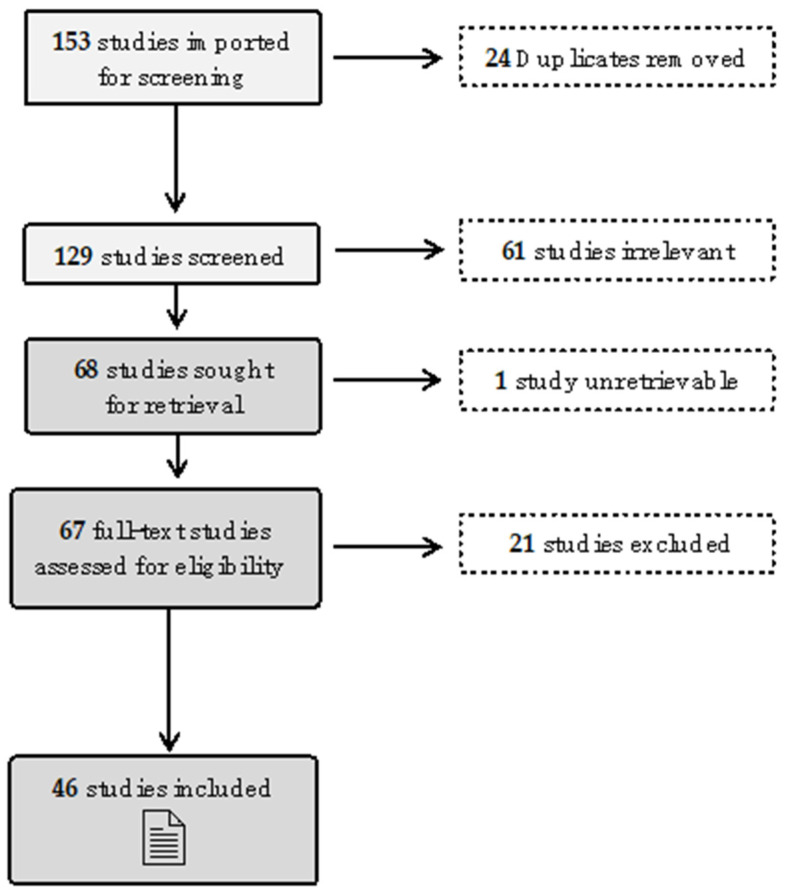
PRISMA Chart.

Figure 1 presents a Preferred Reporting Items for Systematic Reviews and Meta-Analyses (PRISMA) flow chart of articles included in this study:

### 3.1. Rapid Review Literature Analysis Results

Of the total forty-six, one was a policy brief, fourteen were reviews or syntheses and thirty-one were single study papers. Thirty-eight studies focused on the detection of SARS-CoV-2. Four studies discussed the potential to detect other biomarkers (i.e., tuberculosis, mortality related to cardiovascular disease, cancer, diabetes, or chronic respiratory disease, pollution, unsafe water, and lack of sanitation), along with SARS-CoV-2 [30]. One study discussed polio detection [31], and three discussed the detection of illicit and licit substance use [25,30,32].

Thirty-five articles examined low-income and/or resource-constrained settings, ten studies examined rural and/or remote settings, one discussed First Nations communities, and one study examined a Native American reservation. See Appendix A for the table of the included literature.

### 3.2. Sampling Protocols and Sampling Technology

#### 3.2.1. Sampling Technology

In the literature of WBE in rural, remote, or resource-constrained areas, three general sampling technologies are most relevant: grab, composite, and passive samplers. Grab samplers collect samples at a single point in time, whereas composite samplers draw samples, continuously or in smaller increments, over an extended period (usually 24 h) to create a composite sample more representative of “typical” wastewater. Grab samplers are convenient (as they do not require the installation of specialized equipment) and lower in costs but produce fewer representative samples as they can only capture a ‘moment in time’ and may miss significant shedding events [33,34]. This contrasts with composite samplers that are generally higher in cost due to technical features and personnel oversight but can produce a more accurate average of the concentration of the target virus or biomarker as they control for temporal variations (e.g., time-based trends in contribution to wastewater) [35].

Additionally, there are semi-composite samplers that draw samples within a truncated period (lower than the standard 24 h) and can serve as an alternative for sampling sites that are difficult to access or to maintain a composite autosampler for long periods [36]. Wastewater samples from grab samplers can also be manually composited after collection [34]. Samplers are also built with varying degrees of automation, with autosamplers being more technical and costly than non-automatic grab, or passive samplers, which are devices made of absorbent materials, such as cotton buds, Moore swabs, or electronegative filters. Passive samplers can be beneficial as they are easy to operate, cost-efficient, and capable of capturing shedding events as they collect viruses over time [34,37]. However, passive samplers have several drawbacks, the most significant of which is their inability to provide data that accurately measures viral loading in the wastewater (unless combined with a flow meter, which negates their simplicity advantage). Therefore, passive samplers are best suited for presence/absence-type monitoring programs. For non-sewered remote areas, Daigle et al. suggest that while autosamplers are generally preferred, low-cost passive samples may improve access to testing in remote or low-resource contexts [37].

Further, in a comparative review of passive, grab, and composite samplers, Bivins et al. noted that in several studies, passive samplers outperformed grab samplers for detecting SARS-CoV-2 RNA in wastewater and in some instances performed similarly to composite samplers. Citing studies by Schang et al. [38], Habtewold et al. [39], Rafiee et al. [40], and Hayes et al. [41], they summarized that passive sampling using electronegative membranes for exposure duration up to 48 h resulted in ‘promising linear uptake.’ However, due to the indeterminate relationship between flow rate, viral concentration in wastewater, and the degree to which passive samplers can load target viruses, it is not currently possible to use passive sampling to estimate viral concentration consistently [34].

#### 3.2.2. Distribution System Characteristics

The wastewater treatment methods of a given area—whether centralized, decentralized, non-sewered, or informal—greatly impact and shape WBE sampling methodology and protocols. In communities with centralized WWTPs or treatment lagoons, sampling is generally performed at the point of entry to the treatment system. Locations further upstream in the collection network, such as lift or pump stations, or sewer access points, can also be used, depending on the infrastructure and the catchment area of interest. It is worth mentioning that in a study by D’Aoust et al. [42], comparing samples derived from lagoons and pumping stations in a rural community in Eastern Ontario, it was observed that SARS-CoV-2 RNA in lagoon samples was less concentrated and experienced more degradation than samples from pumping stations. The sampling location within a treatment system, such as a lagoon, is an important factor, as increasing degradation of the analyte target (e.g., SARS-CoV-2 RNA) occurs as the wastewater moves through the treatment system. Sampling at the treatment system’s inlet avoids this degradation from the treatment system.

Many studies have shown the proficiency of conducting WBE in regions with centralized WWTPs, while fewer explore the capacity of conducting WBE in regions with decentralized treatment methods or non-sewered systems [8,15,16,43]. This is a critical point, as many rural, remote, low-income, or resource-constrained communities use decentralized facilities or non-sewered wastewater treatment methods, such as discharge to surface waters, septic tanks, or pit latrines [1,9]. In Kolarević et al.’s [44] (p. 5) investigation of WBE of SARS-CoV-2 RNA in the Sava and Danube Rivers of Belgrade, Russia, they report that testing rivers poses complexities not encountered in WWTPs, citing dilution by rain and river waters, as well as animal excrement. They conclude that site selection for river WBE should prioritize smaller surface waters and sites near populated areas “influenced by nearly fresh human sewage and minimally affected by dilution by the river or affected by fecal pollution of animal origin”. Echoing this suggestion, Stockdale et al.’s [26] COVID-19 surveillance study of untreated composite wastewater samples of Nagpur, Central India also found that livestock contributed to WBE findings in rural areas.

In a study on SARS-CoV-2 detection in sewage and river water in a remote and resource-constrained region of Brazil, sample site selection was based on records of inadequate implementation of sanitation protocols, the community’s extensive mining and farming industries, and the location of a regional prison [45]. Barnes et al. [16] conducted a study on SARS-CoV-2 surveillance in Malawi, where formal sewage systems are limited. The study involved sampling from rivers and a non-operational WWTP that received runoff from multiple large rivers. In 2020, seven sites were selected based on previous positive S. Typhi samples, and in 2021, the collection expanded to 112 sites across Blantyre. A GIS framework was used to prioritize locations based on river confluences, population density, and watershed data, with a focus on areas serving the largest population.

In addition to wastewater infrastructure, site selection is largely dependent on a community’s geographic and social-temporal characteristics [9,44]. For example, in a study on WBE for the detection of illicit and licit substances in the wastewaters of an undisclosed Native American reservation with >8000 residents, six individual communities were selected based on their geographic distribution, commercial locations, schools, and healthcare facilities. To avoid sampling older sewage from lagoons, samples were collected within the wastewater collection system rather than the endpoint [32]. Moreover, a study by Jakariya et al. [46] on WBE in developing countries with onsite sanitation facilities investigated the most effective locations for wastewater sampling in 14 districts of Bangladesh that included rural areas. As drainage systems were not available in rural communities, a hotspot-based sampling protocol was implemented in areas with increased transmission risk (such as urban drains at bus and train stations, community ponds, and marketplaces). Cohen et al.’s [19] review of sampling in rural areas of the USA concluded that considering factors such as aging infrastructure, low population density, and diluted pathogen signals, strategies to improve reliability include increasing sampling points, repairing infrastructure, and using advanced detection methods.

Furthermore, WBE approaches themselves can be either centralized (e.g., sampling from a single WWTP or site) or decentralized (e.g., sampling from different points along a sewer system). For communities that rely on decentralized or non-sewered waste management, sampling techniques must be adapted to fit the geographic and social-temporal characteristics of the community [19,46]. This often involves sampling at buildings, septic tanks, surface waters, portable toilets, or pit latrines [30,43,44]. In a 2022 study by Gonçalves et al. [1] on monitoring COVID-19 transmissions, they conclude that using a decentralized approach can be advantageous, not only for regions without centralized WWTPs, but also for assessing the likelihood of outbreaks in small settings like retirement homes, schools, and hospitals.

#### 3.2.3. Sampling Frequency

The frequency of wastewater sampling plays a role in capturing temporal variations for viral shedding in a population. In resource-constrained settings, however, the ability to implement frequent sampling is often limited by logistical and financial constraints. While the majority of sampling protocols involved sample collection at a minimum of twice per week [24,32,44,47,48], lower-frequency schedules may still provide useful public health data. For example, in a study of WBE for SARS-CoV-2 in Addis Ababa, Ethiopia, the sample schedule was set to once per week on Sunday mornings to control for temporal variations in contribution as most community members were home at this time [5]. Likewise, in a WBE study in Indonesia, Murni et al. [49] also concluded sampling once per week or even fortnightly was sufficient for informing public health responses promptly.

Challenges in maintaining regular sampling schedules are often more pronounced in remote or resource-constrained settings. An irregular convenience schedule was implemented in Tandukar et al.’s [34] COVID-19 WBE research in Nepal; however, the authors lamented the lack of a regularized twice-weekly grab sampling schedule due to lockdowns. Renata et al. [48] stated that grab samplers were initially collected once a week but later were reduced to once every two weeks, balancing the need for data with the practical challenges of maintaining more frequent schedules.

In situations where it is necessary to sample bi-weekly or monthly, such as in the polio surveillance in Panama where a monthly grab sampling schedule was used for a year, it is important to note that while this may not be as effective for early detection, it can still provide valuable insights. This schedule meets the minimum recommended frequency for WBE using grab samplers [31]. Sangsanont et al.’s [12] WBE study in Bangkok, Thailand suggested that sampling every 2–3 weeks was adequate for gathering data about the spread of COVID-19; however, they recognize that a more frequent schedule is preferable for using WBE data as an early warning signal.

### 3.3. Laboratory Analysis Protocols

After collection, samples must be kept cool and transported quickly—typically via cold chain courier services—to a laboratory for extraction and analysis. In rural, remote, or resource-limited regions, maintaining a cold chain can be a significant challenge. Once samples arrive at the laboratory, they undergo RNA concentration and extraction, then subsequent analysis using one of many techniques. Table 2, Table 3 and Table 4 present various materials and equipment for SARS-CoV-2 RNA concentration, extraction, and detection, along with key learnings on their accessibility and effectiveness.

Currently, no standardized protocols for SARS-CoV-2 concentration, genomic RNA extraction from wastewater, or subsequent interpretation of results exist [35,51], variability in methodologies may lead to discrepancies. Choosing a protocol depends largely on the equipment and resources available, as well as the experience of the research team involved. For low-resource settings, simpler extraction methods like skimmed milk flocculation, which requires minimal equipment, may be preferable, though they might be less efficient than advanced methods like ultracentrifugation [12,30]. Using silica columns or ultrafiltration requires trained personnel and access to specialized reagents, which may not always be feasible in decentralized laboratories [51]. Additionally, regions with limited access to cold storage or specialized filtration media may find themselves relying on lower-cost and readily available methods, such as adsorption and elution using electropositive filters, despite potential trade-offs in precision and viral recovery rates [12].

Following the detection and quantification of SARS-CoV-2 RNA, data are interpreted with both temporal and spatial resolution to infer trends in the prevalence and distribution of the virus. This interpretation relies on the assumption of a quantitative relationship between SARS-CoV-2 RNA concentration in wastewater and its circulation in the population. However, the nature of transmission is influenced by environmental and biological factors that can complicate interpretations. As a result, the representability of WBE data can vary significantly [8,54]. Despite these uncertainties studies have shown that SARS-CoV-2 RNA signals in wastewater can correlate with clinical data, albeit with varying precision and generally providing lead times of 7 to 24 days. Factors such as sampling frequency, movement patterns of residents, and asymptomatic infections can affect these lead times [12,48]. In a study by de Freitas Bueno et al. [36], the results of WBE in sites located in low-income regions of Brazil were generally higher than those reported in clinical data, but still reflective of infection patterns. They conclude that this imprecision was largely owed to analytical measurement limitations.

Conversely, a study by Meadows et al. [24] in rural areas of Idaho, USA, found that the number of predicted cases informed by WBE results was generally lower than the number of clinically confirmed cases reported. However, their analytical model was able to predict outbreaks ahead of clinically reported cases. Holm et al. [22] state that it may be possible to calibrate wastewater and clinical data in urban areas, but this might not always be feasible in rural or resource-constrained settings. In these situations, it is recommended to establish well-defined detection limits and specific metrics to interpret results that fall below quantification thresholds. This may require adaptive approaches to reporting data with support from regional public health institutions or larger academic audiences.

These results provide insights into the adaptability of WBE technologies and protocols across settings. Communities, particularly those with resource, logistical, and systemic barriers may need to weigh the benefits of using more ‘advanced’ technologies and conducting more frequent sampling (which might necessitate additional resources for troubleshooting) against the feasibility of lower-cost, simpler options like passive samplers.

## 4. Discussion

The well-documented correlation between WBE data trends and clinical observations supports the use of the method to inform public health decisions and emphasizes the importance of addressing challenges to implementing WBE to encourage uptake across diverse contexts [1,7,10,12,13,17,34,35,36,42,43,45,46,47,48,49,55]. Some studies detailing sampling programs in both urban and rural or remote areas have shown that in the latter viral levels of SARS-CoV-2 and the risk of serious illness tended to be higher [15,22].

### 4.1. Perceived Benefits of WBE for Rural, Remote and Resource-Constrained Communities

First Nations reservations in Alberta share characteristics with the rural and/or remote, and resource-constrained communities highlighted in this review. Many lack the healthcare infrastructure to conduct widespread clinical testing for COVID-19 and other infectious diseases. WBE data capture infectivity or prevalence at the population level in real time, accounting for those missed by clinical testing methods. While WBE has been proven as a practical tool for the detection of infectious diseases in a tested population, much of the literature suggests that in communities with logistical challenges and limited resources, it can be used as a primary source of data, owing to its relatively low cost and ability to monitor a large number of people simultaneously [7,8,10,11,12,13,17,18,21,27,32,35,43,47,55]. The method can detect trends in communities that may be affected disproportionately. This was demonstrated in Louisville, where deliberate oversampling in urban areas has revealed disparities in clinical testing rates, indicating the necessity for targeted health resources [23]. However, these suggestions may not be feasible in resource-constrained settings.

Street et al. [56] provided insight into the sub-Saharan African context, maintaining that for low- and middle-income countries where public health systems and human resource capacities are constrained, WBE may prove to be an indispensable strategy for monitoring community-level health. Echoing this sentiment is a study on Davao City, Philippines by Otero et al. [47] who also concluded that not only could WBE be used in regions where clinical testing capacities and healthcare resources are low to support public health decisions, but also to gather community-level data without increasing social stigma for inhabitants. Basu et al.’s [43] study on SARS-CoV-2 RNA detection in a waste-contaminated canal in Bangalore, India, concluded that there is a need for surrogate indicators for mass testing that can assist public health agencies in implementing early responses to infection outbreaks in resource-constrained regions.

Through WBE it is possible to detect viruses and other health indicators that can be geographically linked to a tested population. For instance, through WBE, shedding of the SARS-CoV-2 virus in pre-symptomatic, oligosymptomatic, and asymptomatic individuals can be captured, which otherwise would be missed [5,12,13,14,15,27,30,36,45,47,49]. This is significant as increases in viral RNA in wastewater can be a reliable indicator of impending outbreaks in a community. Many studies stated that upon detection of a spike in viral loads, communities were able to employ public health strategies to reduce further transmissions, such as vaccination encouragement campaigns, mask and physical distancing mandates, and lockdowns [3,7,10,11,12,30,32,36,37,42,43,49,55].

It should be acknowledged that the analytical level of granularity that is possible via WBE is contingent on various factors, including the community’s wastewater infrastructure, population size, work, and migration patterns, the target virus or biomarker, as well as the surveillance and analytical protocols implemented [12,42].

#### Data Exchange, Governance and Sovereignty

Interestingly, despite the significance of WBE data, only two studies captured in this review discussed how WBE protocols were negotiated by stakeholders or how information moved from lab analysis to subsequent public health policy. First, in a study on the feasibility of WBE in low-income and rural communities in Lincoln Parish, Louisiana, Lee et al. [10] discussed the value of collaboration between a local university and public health officials. In this project, undergraduate students received training to participate in the research program, allowing universities to participate in influencing public health policy. Second, Daigle et al. [37] noted how WBE results were translated by the Office of Chief Public Health into public health actions in a trans-Canada study of remote communities.

Considering that the greater research project in which this review is situated is informed by OCAP principles (Ownership, Control, Access, and Possession), we sought to explore the processes of data governance and sovereignty of each study to make inferences about how to best support First Nations with distinct jurisdictional organizations. As few studies discussed who participated in each step of the research process, it was difficult to assess how protocols, including baselines and action thresholds, were negotiated and by whom in any given community. More comprehensive explorations of data governance and sovereignty in these processes WBE are lacking. One exception was the previously discussed study by Driver et al. [32], whereupon formal written approval, WBE research was conducted with the guidance of Tribal Leadership with data from the project owned by the Tribe.

WBE can be used to assess a variety of health determinants in a population, such as substance use [32]. However, data on any health indicator have the potential to be stigmatizing for communities, and there are added ethical concerns about testing in regions with small populations due to the possibility of identifying individuals included in the tested area. As part of their discussion on ethics for environmental scientists engaged in WBE for SARS-CoV-2, Hrudey et al. [4] cite guideline ten of the World Health Organization’s guidelines on ethical issues in public health surveillance, which state that “Governments and others who hold surveillance data must ensure that identifiable data are appropriately secured.” They maintain that those conducting WBE should be cognizant of the sensitivity of public health data which, if mishandled, have the potential to stigmatize communities, breach trust between marginalized communities and public health agencies, or undermine the public’s confidence and hamper the uptake of public health measures to manage the pandemic. To maintain privacy and confidentiality, Gwenzi [8] (p. 8487). urges risk communication to stakeholders and communities engaging in WBE, and the aggregation of data to not identify individual households. In three studies, it was stated that the true parameters of the communities tested were not disclosed to the general population to protect the privacy of community members [31,32,45]. Although identifiable information was withheld, WBE analysis data were still able to inform public health policy, without stigmatizing the community. As WBE expands, particularly in First Nations communities, governance and data exchange mechanisms will be critical to ensure communities can leverage WBE as a public health tool without risking privacy breaches or stigmatization.

### 4.2. Logistical Considerations and Challenges

Logistical considerations significantly impact the feasibility of WBE in rural, remote, and resource-constrained communities. For instance, Driver et al.’s WBE study on an American Native reservation [32] detailed a slew of logistical barriers. It was observed that the connectivity of the reservation to WWTPs (including difficulties in assessing the number of people contributing to lagoons), the use of grab samplers, and targeting compounds prone to degradation during sample collection, transport, and analysis presented unique challenges. Across the literature, we found that major facilitators and barriers to WBE included the following: the cost, precision, and operation of samplers; type of wastewater treatment infrastructure used by the community; and access to specialized laboratories.

#### 4.2.1. Sampler Costs, Precision, and Operation

Though WBE is much less costly and can oversee the general health of a community on a broader scale than widespread clinical testing, it is not without its costs and precision concerns. Further, for rural and remote regions that are less densely populated, the cost of conducting WBE is higher per person than in more densely populated urban areas [9].

Autosamplers are expensive, with costs cited as ranging between $2300 and $7500 USD [34], plus ongoing maintenance or servicing costs. As such, cost can be a major barrier for resource-constrained and low-income regions. The lowest-cost samplers are generally grab samplers that function by collecting a sample at a single time point or passive samplers that use absorption. Because of the ‘snapshot’ mechanism of grab samplers, they cannot account for temporal variation in community contribution to effluent, thus influencing the representability of the sample. On the other hand, passive samplers have varying degrees of precision and uptake potential, due to variations of materials used and site selection. Of note is a novel passive sampler created by researchers at the University of Dalhousie with the ability to detect the SARS-CoV-2 virus in areas with low prevalence. It comprises a 3D-printed spherical cage that houses an absorbent pad, all of which costs $1 CAN per unit [41]. Balancing the costs and usability of samplers with the logistics and characteristics of the region can complicate decision-making. For instance, though autosamplers are technically equipped, they are not only costly, but also require skilled personnel to install, operate, and maintain. In many instances, using a grab sampler might be easier to install and operate and therefore more appropriate. However, attention needs to be paid to how different sampling techniques change how the data can be used (e.g., autosamplers can provide quantitative trends over time, whereas passive samplers can only provide presence/absence indications).

An important determinant in the success of WBE projects is the training of operators to set up samplers and retrieve and store samples. Of the literature included in this review, we found few made reference to operator training or capacity as it pertained to sampling. Rojas-Bonilla et al. [31] mentioned that local personnel were trained to safely collect, handle, and ship samples collected with grab samplers, while Toledo et al.’s [47] WBE study in Northern New England reported that WWTP staff collected composite samples. Insight into sampler operation is valuable, as different samplers require differing degrees of expertise for operation, maintenance, and repair, for which outside support might be required. Further, it is well documented that First Nations Public Works departments are frequently underfunded and understaffed, which can make it difficult for already overburdened water and wastewater operators who can be trained to facilitate WBE [57,58].

#### 4.2.2. Community Wastewater Infrastructure

Inadequate or decentralized wastewater infrastructure was also presented as a key challenge in implementing WBE in low-income and resource-constrained settings [30,46]. Panchal et al.’s [13] overview of sewage surveillance for SARS-CoV-2 in developing countries found that lack of proper sewer systems, leading to inadequate sewerage collection, and the low number and small coverage of treatment plants can make it difficult to estimate the prevalence of viruses in a population. This is pertinent, as decentralized wastewater systems are commonplace in First Nations communities across Canada, often with insufficient oversight or maintenance [59,60]. In some cases, household-level septic systems or open sewage are the only available testing sites, which makes it difficult to collect and process wastewater samples in a standardized manner. An additional challenge to implementing WBE is the absence of sewage network maps. Jakariya et al.’s [46] study in Bangladesh noted that, in rural areas (where most people reside), network maps were often unavailable or ill defined. Likewise, Otero et al.’s [47] WBE study in urbanized, low-income districts of Davao City, Philippines, unserved by WWTPs cited a lack of a sewer-shed map as a major barrier.

#### 4.2.3. Access to Laboratories

Communities engaging WBE must have access to a courier for the transportation of cold samples and to a laboratory capable of concentrating and detecting the SARS-CoV-2 RNA, or the biotarget of interest. Panchal et al. [13] and Street et al. [56] remark that in low-income or developing communities, there is often a lack of analytical services, hindering WBE efforts. These barriers are attributable to the inaccessibility of expensive laboratory equipment and the shortage of skilled laboratory staff for processing and analyzing samples [30,35]. In a trans-Canada study on WBE, Daigle et al. [37] noted that, for many remote Northwest Territories communities, a key barrier to successful implementation was their physical distance from centralized laboratories in Canadian urban centers. Delays arising from sample shipping and processing times countered the potential of data derived from WBE to act as an early warning signal for possible outbreaks. Additionally, not all laboratories are sufficiently experienced or equipped to reliably process and analyze wastewater for genetic markers. Notably, in a review of WBE in low-income settings, Gwenzi [8] identifies a lack of analytical expertise and properly equipped laboratories as potential challenges, emphasizing the value of standardizing laboratories globally to ensure appropriate virus detection, concentration, and quantification.

### 4.3. Equity-Based Implications

Access to healthcare and health-related data is often limited on First Nations, contributing to poor health outcomes for populations. Equity of the WBE method for rural, remote, and resource-constrained communities is contingent on accessibility as well as other socio-political considerations, all of which speak to a need for support from governments and major institutions. The literature suggests that most WBE methods have been implemented in high-income, urban, or peri-urban settings serviced by centralized WWTPs, obscuring the need for cost-efficient methods amendable to smaller, less resourced communities, even though such communities would benefit from its application [1,5,8,12,27,30,34,56]. Further, Sangsanont et al. [12] argued that even though WBE data have been proven to be an effective measure of COVID-19 surges in a community, the method has mostly been implemented in high-income areas, reinforcing socio-economic disparities in public health responses globally. Citing Takeda et al. [61], they maintain that support for resource-constrained communities interested in participating in WBE should involve the engagement of national governments in the legislation of public health, wastewater, environmental water, and outbreak management.

A review by Adhikari et al. [30] on tracking global health indicators as part of the sustainable development goals of the UN explored opportunities and challenges associated with WBE in under-resourced regions. They also argue that while WBE is an extremely valuable tool for lower-income countries, as a result of decentralized WWTPs and the requirement of trained experts, there are major barriers to implementation, which external support could help overcome. de Freitas Bueno et al. [36] highlighted the value of the One Health approach, stating that collaboration between researchers, public health officials, and government agencies enabled WBE in a resource-constrained community of Brazil. Alluding to questions of equity, this study presented community partnerships with research organizations and broader governmental entities as essential for supporting communities with resource-based and logistical barriers.

In resource-constrained areas, the development of sewage surveillance systems relies heavily on stakeholder partnerships and local government collaboration to meet public health needs. In Dhaka, Bangladesh, partnerships with local health officials, public agencies, and international research institutions were key to creating a COVID-19 dashboard that provided timely, accessible data. The tool was iteratively improved based on feedback from stakeholders, addressing the need for clear, visual data representation. Integration of both clinical and sewage data allowed for early detection of infection trends and informed public health decision-making despite resource limitations [62].

Medina et al. [11], in a review of the need for an environmental justice approach for WBE in rural and resource-constrained communities, concluded that a key driver of equity is assessing communities in need of WBE to prioritize new projects. With a focus on ‘Disadvantaged Communities’ (DACs) in California, many of which experienced a high prevalence of COVID-19 infections and a lack of access to other community-level health monitoring methods, they further stipulate that providing increased access to WBE will necessitate innovative sampling technologies, such as sensors, especially in communities without centralized WWTPs. Similarly, Bivins et al. [34] (p. 11) state that “refinement of passive sampling methods and their linkage to a broader environmental surveillance system, in conjunction with environmental laboratory systems, are needed to generate actionable public health data in such settings. Scaling wastewater surveillance to include low-resource settings is vital to secure a more equitable future in WBE”.

#### Socio-Political and Jurisdictional Barriers

In Canada, healthcare and public health are shared responsibilities between federal, provincial, and First Nations governments. First Nations communities often fall under federal jurisdiction for health services, complicating the implementation of WBE systems. This decentralized approach can lead to gaps in funding and policy alignment, particularly when multiple levels of government are involved. In practice, First Nations communities may experience challenges navigating inter-jurisdictional collaborations to secure funding for public health initiatives such as WBE. For example, while the Alberta provincial government allocated $3.4 million for WBE expansions, there are still gaps in infrastructure and service delivery for First Nations [63]. Such funding does not always extend directly to First Nations reserves. Federal government responsibilities toward First Nations’ public health infrastructure have historically been underfunded. This chronic underfunding manifests in disparities in essential services like wastewater treatment facilities. Federal funding allocated to First Nations healthcare initiatives and infrastructure tends to be insufficient to meet the distinct needs of these communities. This insufficient support is apparent when comparing the wastewater systems of urban centers to the often outdated or non-existent systems in rural or remote First Nations communities [64]

The socio-political landscape of First Nations communities is shaped by colonial legacies that have imposed structural and systemic inequities that affect self-determination in health-related decision-making. First Nations leaders must balance the benefits of population-level health monitoring with concerns about privacy and cultural safety. The historical mistrust of government-led health interventions among First Nations communities stems from decades of systemic racism, including discriminatory public health policies, unethical medical experimentation, and ongoing inequities in health care access [65]. The design and implementation of WBE in First Nations communities must prioritize culturally respectful methodologies.

## 5. Conclusions

The current project is part of the growing body of work on multidisciplinary research and collaboration with communities to advance public health initiatives.

The use of WBE for the detection and management of COVID-19 and other health determinants has demonstrated its ability to inform public health decisions globally. Microbiology and community health experts have described WBE as an unbiased method, as it does not divulge sensitive health information of individuals, but rather provides actionable time-sensitive data at the community level [7,24,47]. Because of these strengths, it is effective in providing health indicators for communities that otherwise would have no means of collecting and mobilizing data on a large scale, thus helping to provide health support for medically underserved communities. The relatively low costs of testing, combined with the capability of WBE to test a large number of people at once, make it an attractive tool for diverse communities globally.

This review sought to explore how best rural, remote, and resource-constrained communities can implement WBE, and what barriers to implementation existed. There are a number of unique considerations for rural, remote, and resource-constrained communities to address when participating in WBE, including costs, accessibility, operator capacity, wastewater infrastructure, and subsequent data mobilization. We conclude that by and large, most resource-constrained communities require support from external research and/or governmental bodies to undertake WBE, under the direction of the participating community.

### 5.1. Limitations

This review examined the literature on WBE in rural, remote, and resource-constrained regions to acquire knowledge on implementation, benefits, and challenges that might apply to First Nations in Alberta. As these criteria served as proxies for geographical and economic considerations present in many First Nations, they do not capture nuances relating to cultural, social, and political contexts that must be distinguished when engaging in decolonized, community-centered research.

Rapid reviews, while systematic in nature, can produce different results than systematic reviews [28]. To establish accountability measures, in this project, one reviewer undertook the literature search, two screened abstracts and full texts, and one synthesized literature data. In the case where consensus could not be reached by the two reviewers, studies were sent to a third reviewer for further consideration. Further, iterations of this review were shared across our research team as part of a member-checking approach.

Due to the time-sensitive and applied nature of this field, not all work on WBE in various regions is published in journals or made available in databases. As a result, it is possible that some reports—such as press releases on WBE on First Nations, or in rural, remote, and resource-constrained areas—were not captured in this review.

This review is part of a larger research project comprising a multidisciplinary team with a multitude of perspectives and vantage points. This has shaped the trajectory of this project immensely, allowing us to explore multiple possibilities and inquiries. Though this has largely been a key strength of this work, due to the range of academic and professional backgrounds included on our research team, our exploration and conclusions are transdisciplinary in nature.

### 5.2. Future Research Directions

A part of the larger study is to explore the equity and policy considerations that were largely missing from the literature on WBE in rural, remote, and resource-constrained communities through ongoing engagement with First Nations participating in the pan-Alberta WBE project. We encourage future studies to explore equity regarding environmental surveillance and how communities can best be supported to engage in WBE and mobilize data for public health policies. Additionally, reflecting on the dearth of studies that present the exchange of data between different stakeholders and institutions that ultimately inform public health policy, we hope to explore these interactions and encourage future studies to do the same.

## Figures and Tables

**Table 1:** Search Terms.

Setting terms	First Nation *	OR “reservation *” OR “Indigenous” OR “American Native”
	Remote and/or Rural	OR “isolated” OR “country *”
	Resource-constrained	OR “low income” OR “refugee” OR “medically underserve*” OR “underserve *” OR “At risk”
Method terms	Wastewater-based epidemiology	OR “WBE” OR “Wastewater testing” OR “wastewater-based monitoring” OR “wastewater surveillance” OR “WWS” OR “Wastewater analysis” OR “WWA” OR “WWW-BE” OR “Sewage”
Topic terms	COVID-19	OR “SARS-CoV-2” OR “Hepatitis *” OR “HVC” OR “Polio” OR “epidemic” OR “pandemic” OR “infectious disease” OR “communicable disease” OR “disease transmission” OR “Infectious disease”

An asterisk “*” signifies that the search results include different forms of the root word.

**Table 2:** SARS-CoV-2 RNA Concentration Techniques and Considerations.

Procedure	Considerations
Process samples for SARS-CoV-2 RNA concentration Key Techniques/Methods: Adsorption and elution using electropositive filters Aluminum Polychloride Flocculation Calcium Flocculation-Citrate Dissolution (CFCD) Nanotrap^®^ Magnetic Beads (NMBs) Polyethylene Glycol (PEG) Precipitation Porcine Gastric Mucin-conjugated Magnetic Beads Silica Columns Skimmed Milk Flocculation (SM) Solid Fraction Extraction Ultracentrifugation Glass Wool Filtration Ultrafiltration	Protocol selection depends on cost, available equipment, and feasibility, which vary widely in resource-constrained settings. Method selection significantly impacts sensitivity. Zhu et al. [50] highlight Skimmed Milk Flocculation (SMF) for significantly improving ZIKV RNA recovery and offering advantages in resource-constrained settings. It is low cost, can be done in less than a day, and requires only standard laboratory equipment. Mainardi and Bidoia report high costs for CentriconPlus-70 centrifugal filters (USD $30–$40 per filter), limiting accessibility in lower-income regions [51]. Wehrendt et al. [52] present a rapid and accessible protocol for concentrating SARS-CoV-2 RNA that costs less than USD $0.01 per sample. Salvo et al. [53] compare the sensitivity of three concentration methods: PEG precipitation, SMF, and aluminum polychloride flocculation. They found PEG precipitation to be the most sensitive method for viral concentration from wastewater, outperforming SMF and aluminum polychloride flocculation. Barbosa et al. [6] evaluate ultracentrifugation with glycine versus Centricon^®^ Plus-70 ultrafiltration and found ultracentrifugation more sensitive and efficient for quantifying SARS-CoV-2 RNA. Banadaki et al. [20] highlight SMF as an affordable, efficient option costing less than USD $2 for four replicates and requiring minimal equipment, completing within 45 min. PEG precipitation, while sensitive, is more expensive and requires up to six hours and advanced equipment.

**Table 3:** RNA Extraction Kits and Resource Accessibility.

Procedure	Considerations
RNA Extraction Commonly used kits: RNeasy PowerMicrobiome Kit (QIAGEN, Hilden, Germany) QIAamp^®^ Viral RNA Mini kit (QIAGEN, Hilden, Germany) Other kits: Dionex™ AutoTrace™ 280 Solid-Phase Extraction Instrument (Thermo Scientific, Waltham, MA, USA) High Pure Viral Nucleic Acid Kit (Roche, Basel, Switzerland) Illumina TruSeq Total RNA Library Preparation Kit (Illumina, San Diego, CA, USA) MagaPure Nucleic Acid Extraction and Purification Kit (BIGFISH, Biological Technology, San Diego, CA, USA) MagMax Viral/Pathogen Nucleic Acid Isolation Kit (Thermo Fisher Scientific, Waltham, MA, USA) Promega WW TNA Capture Kit PureLink Viral RNA/DNA Mini Kit (Thermo Fisher Scientific) QIAcube Connect (QIAGEN, Hilden, Germany) Qiagen QIAamp MinElute Virus Kit	Zhu et al. [50] compared two extraction kits in a low-resource setting: Qiagen QIAamp MinElute Virus Kit and Qiagen RNeasy PowerMicrobiome Kit. The latter, with its bead-beating step, performed better with solids-rich samples, while the MinElute Virus Kit worked well for supernatant samples. Cost of kits ranges from approximately CAD $6.50 to CAD $24.00 per prep, depending on the brand.

**Table 4:** Detection and Quantification Methods for SARS-CoV-2 RNA.

Procedure	Considerations
Detection and Quantification Techniques Standard techniques: The detection and quantification of the SARS-COV-2 virus and other infectious diseases are performed in the lab using the same Polymerase Chain Reaction (PCR) based techniques that are standard in clinical testing. This includes PCR and RT-PCR for basic detection, as well as quantitative PCR (qPCR) and reverse transcription qPCR (RT-qPCR) for quantification, which amplify specific viral genetic material to determine its presence and concentration. Kits used for PCR-based tests: 2019-nCoV RUO Kit (IDT, IA, Carlsbad, CA, USA) AllplexTM 2019 nCoV Assay (Seegene Inc., Seoul, Republic of Korea) GT-Digital SARS-CoV-2 Wastewater Surveillance Assay for QIAcuity^®^ (GT Molecular, Fort Collins, CO, USA) QIAcuity Digital PCR System (QIAGEN, Germantown, MD, USA) TaqPathTM 1-Step RT-qPCR Master Mix, CG (ThermoFisher, Waltham, MA, USA) VetMAXTM XenoTM Internal Positive Control VIC Assay Xeno RNA Control (Applied Biosystems, Thermo Fisher Scientific, Waltham, MA, USA)	Assays are specific tests used to detect and quantify the presence of SARS-CoV-2 RNA in samples; they can be included in kits or acquired separately. Tandukar et al.’s [34] examination of SARS-CoV-2 RNA detection in wastewater, river water, and hospital wastewater in Nepal using grab samplers found the CDC-N2 assay to have the highest sensitivity compared to four other qPCR assays (CDC-N1, NIID_2019-nCOV_N, and N_Sarbeco). Systems to perform assays must also be selected. Daigle et al. [37] noted that there are currently no rapid, sensitive, mobile wastewater tests for SARS-CoV-2. They demonstrated that while the GeneXpert cartridge-based rapid molecular clinical test system could detect SARS-CoV-2 in wastewaters, it only worked when the SARS-CoV-2 abundance was above 32 copies/mL and could not detect variants. Donia et al. [7] suggested that recent advancements in microfluidic technology present a cost-effective approach for SARS-CoV-2 detection, providing opportunities for improved monitoring in various settings.

## Appendix A

**Table A1:** Inventory of WBE studies with discussion of settings with logistical challenges included in the rapid review.

First Author (Year)	Article Type	Target	Setting Category	Area(s) of Focus
Acheampong [15] (2023)	Single Study	SARS-Cov-2 Virus	Low Income and/or Resource-constrained Setting(s); Rural and/or Remote Setting(s)	Nupur, India
Adhikari [30] (2022)	Literature Review	SARS-Cov-2 Virus, illicit and licit substance use, and the potential to detect various biomarkers	Low Income and/or Resource-constrained Setting(s)	Various
Ali [5] (2022)	Single Study	SARS-Cov-2 Virus	Low Income and/or Resource-constrained Setting(s)	Addis Ababa, Ethiopia
Barbosa [6] (2022)	Single Study	SARS-CoV-2 Virus	Low Income and/or Resource-constrained Setting(s)	Sao Paulo, Brazil
Barnes [16] (2023)	Single Study	SARS-CoV-2 Virus	Low Income and/or Resource-constrained Setting(s)	Blantyre and Lilongwe, Malawi
Basu [43] (2022)	Single Study	SARS-CoV-2 Virus	Low Income and/or Resource-constrained Setting(s)	Bengaluru (Bangalore), India
Bivins [34] (2022)	Literature Review	SARS-Cov-2 Virus	Low Income and/or Resource-constrained Setting(s)	Various
Belmonte-Lopes [17] (2023)	Single Study	SARS-Cov-2 Virus	Low Income and/or Re-source-constrained Setting(s)	Curitiba, Southern Brazil
Cancela [18] (2023)	Single Study	SARS-Cov-2 Virus	Low Income and/or Re-source-constrained Setting(s)	Montevideo, Salto, Rivera, Castillos, and Melo, Uruguay
Cohen [19] (2023)	Literature Review	SARS-CoV-2 Virus, Norovirus GII, and Pepper mild mottle virus	Rural and/or Remote Setting(s); Low Income and/or Re-source-constrained Setting(s)	Various areas of the United States of America
D’Aoust [11](2021)	Single Study	SARS-Cov-2 Virus	Rural and/or Remote Setting(s)	A small sewered community (less than 5000 inhabitants) in Eastern Ontario
Daigle [37] (2022)	Single Study	SARS-Cov-2 Virus	Rural and/or Remote Setting(s)	5 communities in the Northwest Territories (Yellowknife, Hay River, Inuvik, Fort Smith, and Fort Simpson)
de Araujo [35] (2021)	Literature Review	SARS-CoV-2 Virus	Low Income and/or Resource-constrained Setting(s)	Various
Banadaki [20] (2024)	Single Study	SARS-CoV-2 Virus	Low Income and/or Resource-constrained Setting(s)	9 WWTPs in eastern Kentucky, USA
de Freitas Bueno [36] (2022)	Single Study	SARS-Cov-2 Virus	Low Income and/or Resource-constrained Setting(s)	Sao Paulo and Foz do Iguau, Brazil
Donia [7] (2021)	Literature Review	SARS-Cov-2 Virus	Low Income and/or Resource-constrained Setting(s)	Various
Driver [32] (2022)	Single Study	Licit and illicit substances	First Nations/Native American Reservation(s)	Undisclosed reservation located in the United States (population > 8000 individuals)
Dzinamarira [28] (2022)	Literature Review	SARS-CoV-2 Virus	Low Income and/or Resource-constrained Setting(s)	Africa (various locales)
Fongaro [45] (2021)	Single Study	SARS-Cov-2 Virus	Low Income and/or Resource-constrained Setting(s)	Minas Gerais, Brazil
Gonçalves [1] (2022)	Literature Review	SARS-CoV-2 Virus	Low Income and/or Resource-constrained Setting(s)	Various
Gwenzi [8] (2022)	Literature Review	SARS-Cov-2 Virus	Low Income and/or Resource-constrained Setting(s)	Various
Hassard [21] (2023)	Literature Review	SARS-CoV-2 Virus, Influenza, and the potential to detect other infectious diseases	Low Income and/or Resource-constrained Setting(s)	Various
Holm [22] (2023a)	Literature Review	SARS-CoV-2 Virus	Low Income and/or Resource-constrained Setting(s); Rural and/or Remote Setting(s)	South Africa, United States of America,
Holm [23] (2023b)	Single Study	SARS-CoV-2 Virus	Low Income and/or Re-source-constrained Setting(s)	Louisville, Kentucky; Houston, Texas
Hrudey [9] (2022)	Policy Brief	SARS-CoV-2 Virus	Various, including Rural and/or Remote Setting(s)	Canada
Jakariya [46] (2022)	Single Study	SARS-Cov-2 Virus	Low Income and/or Resource-constrained Setting(s)	Bangladesh
Kolarevic [44] (2022)	Single Study	SARS-Cov-2 Virus	Low Income and/or Resource-constrained Setting(s)	Belgrade, Russia (The Sava River, and the Danube River)
Lee [10] (2022)	Single Study	SARS-Cov-2 Virus	Rural and/or Remote Setting(s)	Lincoln Parish, Louisiana (City of Ruston and City of Grambling)
Meadows [24] (2023)	Single Study	SARS-Cov-2 Virus	Rural and/or Remote Setting(s)	Various rural communities in Idaho, United States of America
Medina [11] (2022)	Literature Review	SARS-Cov-2 Virus	Low Income and/or Resource-constrained Setting(s); Rural and/or Remote Setting(s)	Disadvantaged Communities (DAC) in California
Murni [49] (2022)	Single Study	SARS-Cov-2 Virus	Low Income and/or Resource-constrained Setting(s)	Special Region of Yogyakarta Province, Indonesia
Oertel [25] (2023)	Single Study	Illicit substances	Rural and/or Remote Setting(s)	15 German WWTPs of varied sized, including those serving rural areas.
Otero [47] (2022)	Single Study	SARS-CoV-2 Virus	Low Income and/or Resource-constrained Setting(s)	Davao City, Philippines
Panchal [13] (2021)	Literature Review	SARS-Cov-2 Virus	Low Income and/or Resource-constrained Setting(s)	Various (India, Africa, Bangladesh, Afghanistan, Nepal, Myanmar and Indonesia)
Pandey [14] (2021)	Literature Review	SARS-CoV-2 Virus	Low Income and/or Resource-constrained Setting(s)	Developing countries (non-specified)
Rojas-Bonilla [31] (2021)	Single Study	Polioviruses	Low Income and/or Resource-constrained Setting(s)	Las Mendozas (La Chorrera), Villa Real (La Chorrera), David (Chiriquí), Las Lomas (Chiriquí), Nuevo Tocumen (Panama City), Panama
Salvo [53] (2021)	Single Study	SARS-Cov-2 Virus	Low Income and/or Resource-constrained Setting(s)	Salto, Uruguay
Sangsanont [12] (2022)	Single Study	SARS-Cov-2 Virus	Low Income and/or Resource-constrained Setting(s)	Bangkok, Thailand
Stockdale [26] (2023)	Single Study	SARS-Cov-2 Virus	Rural and/or Remote Setting (s)	Nagpur district, Central India
Street [56] (2020)	Literature Review	SARS-CoV-2 Virus	Low Income and/or Resource-constrained Setting(s)	Africa (sub-Saharan)
Tandukar [33] (2022)	Single Study	SARS-CoV-2 Virus	Low Income and/or Resource-constrained Setting(s)	Kathmandu Valley, Nepal
Toledo [55] (2022)	Single Study	SARS-CoV-2 Virus	Rural and/or Remote Setting(s)	Northern New England
Wardi [27] (2024)	Single Study	SARS-CoV-2 Virus, Influenza A and B, and RSV	Low Income and/or Resource-constrained Setting(s)	Agadir and Inezgane, Morocco
Wehrendt [52] (2021)	Single Study	SARS-Cov-2 Virus	Low Income and/or Resource-constrained Setting(s)	Buenos Aires Metropolitan Area, Argentina
Wettstone [62] (2023)	Single Study	SARS-Cov-2 Virus	Low Income and/or Re-source-constrained Setting(s)	Dhaka, Bangladesh
Zhu [50] (2023)	Single Study	Zika Virus	Low Income and/or Resource-constrained Setting(s)	Salvador, Bahia, Brazil
